# Breast metastasis of gastric signet-ring cell carcinoma: a case report and literature review

**DOI:** 10.1186/s12957-015-0538-1

**Published:** 2015-03-26

**Authors:** Chun-Lan He, Ping Chen, Bing-Lan Xia, Qin Xiao, Feng-Lin Cai

**Affiliations:** Department of Thyroid and Breast Surgery, Subei People’s Hospital, Yangzhou University, No. 98 Nantong West Road, Yangzhou, 225001 Jiangsu Province China; Department of Gastrointestinal Surgery, Subei People’s Hospital, Yangzhou University, No. 98 Nantong West Road, Yangzhou, 225001 Jiangsu Province China; Department of Ultrasonography, Subei People’s Hospital, Yangzhou University, No. 98 Nantong West Road, Yangzhou, 225001 Jiangsu Province China; Department of Pathology, Subei People’s Hospital, Yangzhou University, No. 98 Nantong West Road, Yangzhou, 225001 Jiangsu Province China

**Keywords:** Mammary carcinoma, Metastasis, Gastric adenocarcinoma, Signet-ring cell, Immunohistochemistry

## Abstract

**Background:**

Cases of primary gastric adenocarcinoma with metastasis to the breast are extremely rare. Till now, only 38 cases have been reported in PubMed since 1908.

**Case presentation:**

We herein reported a race case of gastric adenocarcinoma with metastasis to the right breast. Breast biopsy showed invasive signet-ring cell breast carcinoma in the right breast. She was given a TEC regimen (docetaxel 75 mg/m^2^, epirubicin 75 mg/m^2^, and cyclophosphamide 600 mg/m^2^ every 3 weeks) for one cycle but showed no objective response. Upper gastrointestinal endoscopy demonstrated an ulcerative mass in the gastric body. Biopsy demonstrated low-grade gastric adenocarcinoma with signet-ring features. In immunohistochemistry, mammary malignant cells were positive for cytokeratin 7, cytokeratin 20, villin, and ErbB2/HER2, but negative for gross cystic disease fluid protein-15, estrogen receptor, and progesterone receptor. The diagnosis of metastatic poorly differentiated signet-ring cell adenocarcinoma of the right breast identical to gastric primary was confirmed finally.

**Conclusions:**

Gastric cancer with metastasis to the breast can be diagnosed by clinical history, histological findings, and immunohistochemical markers.

## Background

Gastric carcinoma with metastasis to the breast is extremely rare. Only 38 cases have been reported in PubMed thus far. The lymph node dissemination might be the possible mechanism of metastasis from the stomach to the breast. Sometimes, a metastatic tumor in an occult site may be difficult to be distinguished between a synchronous or metachronous primary cancer and a metastatic disease, especially when it is asymptomatic. In this study, we reported a case of a 48-year-old Chinese woman with a metastasis to the right breast from a gastric signet-ring cell carcinoma and reviewed the literature.

## Case presentation

**Figure 1:**
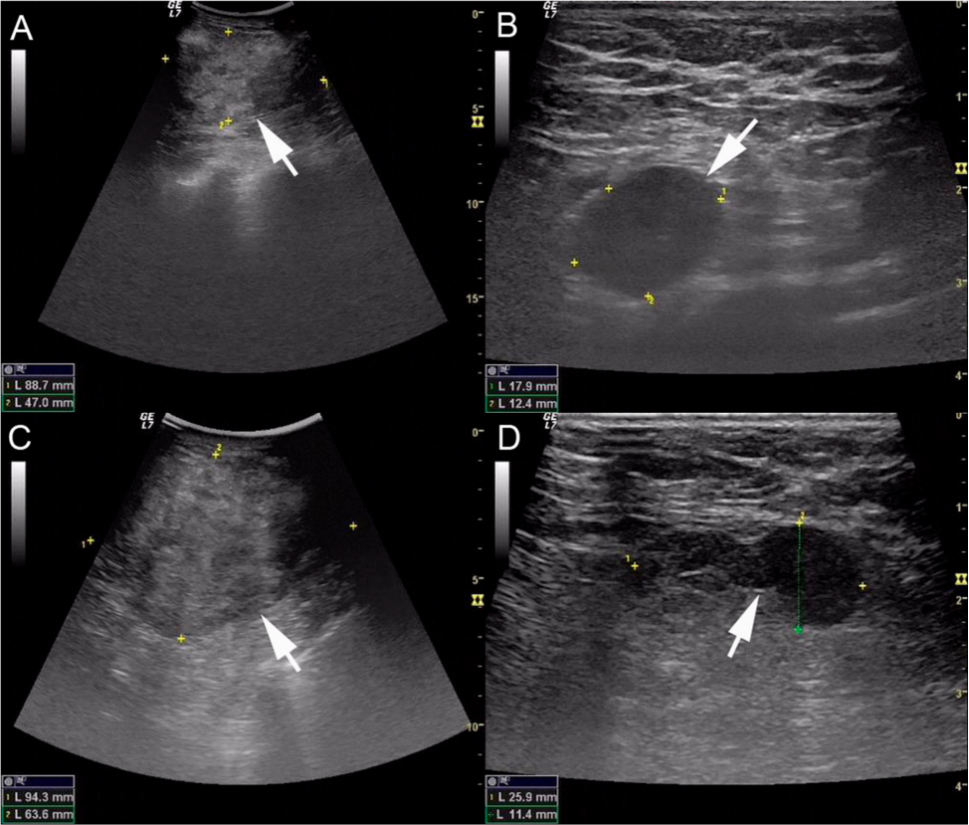
**Ultrasonography of the breast.** In 30 July 2014, ultrasonography revealed an 88.7-mm × 47.0-mm ill-defined heterogeneity lesion (arrow) in the upper inner quadrant of the right breast **(A)** with right enlarged axillary lymph nodes (arrow), 17.9 mm × 12.4 mm in diameter **(B)**. In 21 August 2014, ultrasonography revealed a 94.3-mm × 63.6-mm ill-defined heterogeneity lesion (arrow) in the upper inner quadrant of the right breast **(C)** with right enlarged axillary lymph nodes (arrow), 25.9 mm × 11.4 mm in diameter **(D)**.

**Figure 2:**
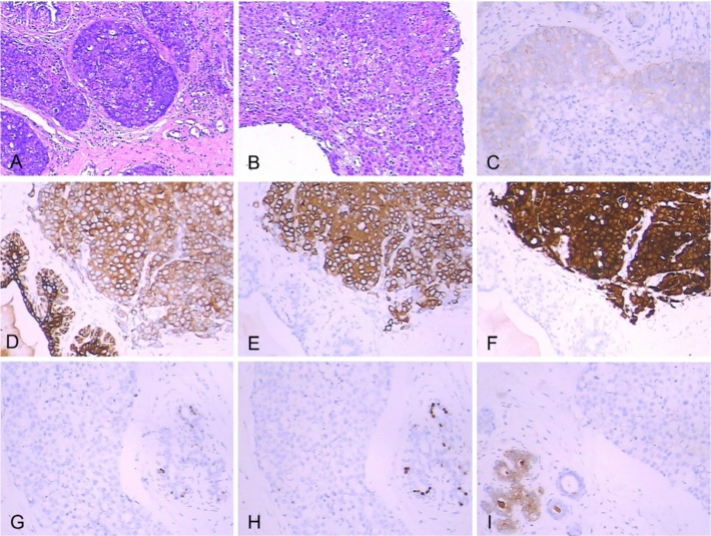
**Breast and gastric biopsy and immunohistochemical analysis.** Breast biopsy showed invasive carcinoma with signet-ring cells (hematoxylin and eosin, magnification × 100) **(A)**. Gastric biopsy showed infiltration from a diffuse-type low-grade gastric adenocarcinoma with signet-ring features (hematoxylin and eosin, magnification × 100) **(B)**. Immunohistochemical analysis revealed mammary tumor cells were positive for ErbB2/HER2 **(C)**, CK7 **(D)**, CK20 **(E)**, and villin **(F)**, but negative for ER **(G)**, PR **(H)**, and GCDFP-15 **(I)** (3,3′-diaminobenzidine, magnification × 100).

A 48-year-old Chinese woman was admitted to the Subei People’s Hospital of Jiangsu Province, China, on 29 July 2014. She complained of a lump in the right breast. Physical examination showed an 8.0-cm × 5.0-cm mass lying in the upper inner quadrant of the right breast with axillary lymphadenopathy on both sides. The ultrasound showed an 8.9-cm × 4.7-cm ill-defined lesion in the upper inner quadrant of the right breast and a 1.8-cm × 1.2-cm enlarged lymph node in the right axilla (Figure 1A,B). Core needle biopsy showed invasive signet-ring cell breast carcinoma (Figure 2A). Immunohistochemistry (IHC) showed that tumor cells were positive for epidermal growth factor receptor (EGFR) and ErbB2/HER2, but negative for estrogen receptor (ER) and progesterone receptor (PR). Serum tumor markers including carcinoembryonic antigen (CEA), cancer antigen (CA) 153, CA125, and CA199 did not elevate. A TEC regimen (docetaxel 75 mg/m^2^, epirubicin 75 mg/m^2^, and cyclophosphamide 600 mg/m^2^ every 3 weeks) was administered as neoadjuvant chemotherapy.

**Figure 3:**
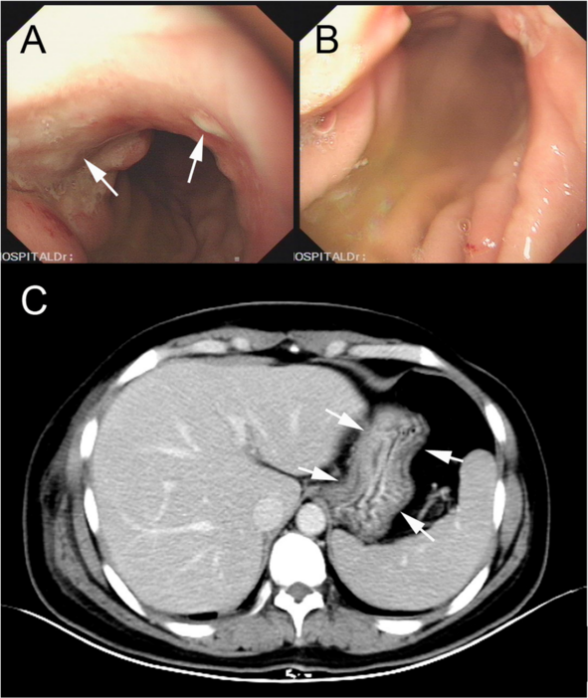
**Gastric endoscopy and enhanced abdominal CT scan.** Gastric endoscopy showed an ulcerative mass in the gastric body (arrows) **(A,B)**. Enhanced abdominal CT scan revealed a circumferential mural thickening of the gastric body wall (arrows) **(C)**.

Because of less response to chemotherapy, ultrasonography was performed and showed an increased 9.4-cm × 6.4-cm ill-defined hypoechoic mass in the upper inner quadrant as well as a 2.6-cm × 1.1-cm enlarged lymph node in the right axilla on 21 August 2014 (Figure 1C,D). Enhanced abdominal computed tomography (CT) revealed a circumferential mural thickening of the gastric body wall (Figure 3C). Upper gastrointestinal endoscopy demonstrated an ulcerative mass in the gastric body (Figure 3A,B). Biopsy of the lesion revealed infiltration from a diffuse-type low-grade gastric adenocarcinoma with signet-ring features (Figure 2B). Serum tumor markers including CEA, CA153, CA125, and CA199 were measured, and only CA199 was highly elevated (more than 1000 IU/mL). Further immunohistochemistry showed the tumor was positive for cytokeratin 7 (CK7), CK20, villin, and ErbB2/HER2, but negative for gross cystic disease fluid protein-15 (GCDFP-15), ER, and PR (Figure 2C,D,E,F,G,H,I and Table 1). These features helped to make the diagnosis of primary gastric adenocarcinoma with metastasis to the right breast. Then, the patient was treated with SOX regimen for four cycles (S-1 80 mg/m^2^, oxaliplatin 100 mg/m^2^). At time of submission of our manuscript, the patient responded well to the adjusted chemotherapy and was followed for 4 months after the definite diagnosis was made.

**Table 1:** **Immunohistochemical analysis of mammary tumor cells**

**Antibodies**	**P/N**	**Antibodies**	**P/N**
CK7	+	GST-Π	++
CK20	+	Ki-67 labeling index	60%
EGFR	+	PR	−
ER	−	P-gp	−
ErbB2/HER-2	+	TOPO-II	++
GCDFP-15	−	Villin	+

P/N: positive/negative; CK: cytokeratin; EGFR: epidermal growth factor receptor; ER: estrogen receptor; ErbB2/HER-2: human epidermal growth factor receptor 2; GCDFP-15: gross cystic disease fluid protein-15; GST-Π: glutathione S transferases Π; PR: progesterone receptor; P-gp: P-glycoprotein; TOPO-II: topoisomerase II; −: no cells positive by IHC; ±: sometimes weak positive, sometimes negative by IHC; +: <25% of cells positive by IHC; ++: 25%–50% of cells positive by IHC; +++: >50% of cells positive by IHC.

## Discussion

Metastases from extra-mammary malignant neoplasms are rare, constituting only 0.5% to 2.0% of all mammary malignancies [1]. Malignant melanoma, lymphoma, lung cancer, ovarian cancer, and soft tissue sarcoma have been reported as the most common original tumor of mammary metastases. Gastrointestinal and genitourinary tumors are less common primary sites. Sporadic cases of mammary metastasis have been reported from osteosarcoma, thyroid neoplasm, and cervical, vaginal, and endometrial carcinoma [2-6].

Since primary gastric adenocarcinoma with metastasis to the breast is extremely rare, PubMed, MEDLINE, Embase, and Google Scholar were searched till September 2014 using the key words such as “gastric or stomach”; “tumor or cancer or carcinoma or adenocarcinoma”; “breast or mammary”; and “metastasis.” Only 38 cases have been reported previously. We made a summary of all these 38 cases as well as the present case to highlight their clinicopathological characteristics (Table 2). The age of these patients ranged from 23 to 70 years (mean 46.5 years; median 46 years). Among 36 cases, 22 harbored a histological feature of signet-ring cell adenocarcinoma. Moreover, multiple metastases could be found in these cases except the breast.

**Table 2:** **Clinicopathological features of reported cases of primary gastric carcinoma with breast metastasis**

**Case [ref]**	**Age**	**Side**	**Differentiation**	**Interval (mo)**	**Other metastases**	**Follow-up (mo)**
Reitmann *et al*. 1908^a^	33	R + L	Scirrhous	–	–	–
Kreibich *et al*. 1909^a^	65	R	Scirrhous	–	Skin	–
Mourier *et al*. 1910^a^	31	L	Mucinous	–	Liver and pancreas	–
Stahr *et al*. 1922^a^	46	R + L	Anaplastic	–	–	–
Dawson *et al*. 1936^a^	25	R + L	Mucinous	–	Ovaries	–
Abrams *et al*. 1949^a^	–	–	–	–	–	–
Sandison *et al*. 1959^a^	56	L	Signet-ring cell	–	–	–
[14]	59	R + L	–	4	Axillary lymphadenopathy	6
Hajdu *et al*. 1972^a^	–	L	Adenocarcinoma	–	–	–
Silverman *et al*. 1974 [15]	–	–	Mucin-producing	0	–	–
Toombs *et al*. 1977^a^	–	–	–	–	–	–
Satake *et al*. 1980^a^	39	L	Signet-ring cell	0	–	–
Togo *et al*. 1980^a^	70	L	Signet-ring cell	0	–	–
Nielsen *et al*. 1981^a^	59	L	Mucinous	0	–	–
Champault *et al*. 1982^a^	65	L	Adenocarcinoma	0	–	–
[16]	46	L	Signet-ring cell	0	Axillary lymphadenopathy	12 days
Kasuga *et al*. 1986^a^	48	R + L	Signet-ring cell	31	–	–
[17]	28	R + L	Mucinous differentiation	0	Lymph nodes	–
[18]	31	R	Signet-ring cell	0	Lymph nodes, ovaries	–
[19]	36	L	Poorly with signet-ring cells	72	Axillary lymphadenopathy	3
[20]	–	–	Signet-ring cell	–	–	–
Domanski *et al*. 1996 [21]	48	L	Signet-ring cell	0	Left supraclavicular nodes	–
de la Cruz Mera *et al*. 1998 [22]	61	L	Signet-ring cell	13	Pleura	–
[23]	46	R + L	Signet-ring cell	2	Bilateral axillary nodes	–
[24]	41	L	Signet-ring cell	0	Ovaries	–
[24]	23	R	Signet-ring cell	0	Axillary nodes	–
Madan *et al*. 2002 [10]	39	R	Signet-ring cell	3	Ovaries, peritoneum	–
[25]	39	R + L	Signet-ring cell	1	Ovaries, peritoneum, skin	–
Boutis *et al*. 2005 [12]	37	L	Signet-ring cell	0	Ovaries	6
[26]	37	L	Poorly	0	–	6
[27]	61	R	Poorly	48	Peritoneum	2
Makni *et al*. 2007 [7]	40	R	Signet-ring cell	4	Ovaries, spleen	18
[11]	67	L	Poorly with signet-ring cells	5	Left axillary and supraclavicular nodes	4
Cil *et al*. 2009 [28]	63	L	Signet-ring cell	12	Left axillary nodes	4
Cil *et al*. 2009 [28]	65	L	Signet-ring cell	24	Right ovarian, liver and lung	6
Soler *et al*. 2010 [9]	37	L	Signet-ring cell	2	Ovarian	7
[29]	37	L	Signet-ring cell	31	-	-
[30]	54	R	Signet-ring cell	0	Right ovarian	11
This case	48	R	Signet-ring cell	0	Right axillary nodes	2 alive

^a^References are included in [18]. Interval: the time between the diagnosis of gastric carcinoma and the development of metastasis to the breast; mo, month; Poorly: poorly differentiated adenocarcinoma; Signet-ring cell: signet-ring cell carcinoma.

Metastatic mammary carcinoma is usually correlated with a poor prognosis. Based on the previous reported cases, the overall survival lasted from 12 days to 18 months. In the metastatic process, mammary involvement could either be the first station or occur in a polymetastatic context [7]. Although the pathway by which malignancies metastasized to the breast remains unknown, Vergier *et al*. [8] hypothesized that estrogen may play a role in promoting extra-mammary tumorigenesis. Also, abundant blood supply of the breast has been proposed as the mechanism for the increased incidence of breast metastasis in premenopausal women. On the other hand, the metastases from gastric carcinoma to the breast had a surprisingly unified tendency. The left breast involved accounted for 55.9% (19/34) of all cases, while the right side accounted for 23.5% (8/34) and both sides 20.6% (7/34). Accordingly, Parrell *et al*. [9] reported that breast metastases were most commonly found in the upper outer quadrant of the left side. This phenomenon suggested the left supraclavicular lymph node might be important in the process of metastasis from gastric carcinoma to the breast. Occult adenocarcinoma often poses a challenge to clinicians and pathologists and may lead to an absolutely different therapeutic strategy. Histopathology is useful to differentiate mammary metastasis from primary breast cancer [10]. IHC remains the main choice in identifying the tumor origin. Although only few of tumor markers are very specific with limited sensitivity, they can be used as a panel to improve the sensitivity. IHC staining for breast metastasis from gastric cancer is usually negative for ErbB-2, ER, PR, and GCDFP-15, but positive for epithelial markers like CEA, CK7, and CK20 [11-13].

## Conclusions

A primary gastric adenocarcinoma with metastasis to the breast is an extremely rare malignancy and is usually associated with poor prognosis. Clinical history, histological findings, and immunohistochemical markers such as CK20, CK7, CDX-2, villin, and GCDFP-15 are helpful in distinguishing primary breast cancer from breast metastasis of gastric cancer.

## Consent

Written informed consent was obtained from the patient.
